# Supplementary material for: Assessing the impact of apnea duration on the relationship between obstructive sleep apnea and hearing loss
Source: PLoS One. 2024 Dec 18;19(12):e0315580. doi: 10.1371/journal.pone.0315580 (PMC11654924; doi:10.1371/journal.pone.0315580)
Supplement: Attachment — Submitted filename: Response to Reviewers.docx [file pone.0315580.s002.docx]

**Changes of references**

Removed list: none

Added list: #3, 4, 5, 27 (All references were added to incorporate the reviewer’s suggestions.)

3. Iannella G, Magliulo G, Greco A, de Vincentiis M, Ralli M, Maniaci A, et al. Obstructive sleep apnea syndrome: From symptoms to treatment. Int J Environ Res Public Health. 2022;19: 2459. doi: [10.3390/ijerph19042459](https://doi.org/10.3390/ijerph19042459).

4. Oksenberg A, Leppänen T. Duration of respiratory events in obstructive sleep apnea: In search of paradoxical results. Sleep Med Rev. 2023;68: 101728. doi: [10.1016/j.smrv.2022.101728](https://doi.org/10.1016/j.smrv.2022.101728).

5. Peppard PE, Young T, Barnet JH, Palta M, Hagen EW, Hla KM. Increased prevalence of sleep-disordered breathing in adults. Am J Epidemiol. 2013;177: 1006–1014. doi: [10.1093/aje/kws342](https://doi.org/10.1093/aje/kws342).

27. Ekin S, Turan M, Arısoy A, Gunbatar H, Sunnetcioglu A, Asker S, et al. Is there a relationship between obstructive sleep apnea (OSA) and hearing loss? Med Sci Monit. 2016;22: 3124. doi: 10.12659/msm.897347.

**Response to reviewers**

**Reviewer #1:**

Introduction:

1. Give additional background on the clinical significance of investigating the connection between OSA and hearing loss.

Thank you for your comments. We have modified the paragraph as follows.

(Lines 51-53) However, despite the growing prevalence of OSA, comprehensive data on whether hearing level changes occur in patients with OSA and the patterns these changes follow remain scarce. This gap highlights the need for further investigation to elucidate the auditory effects of OSA.

2. Clearly state why the length of apnea is a significant aspect. cite doi:10.3390/ijerph19042459

Thank you for your comments. We have modified the paragraph and cited the recommended article.

(Lines 45-50) Airway blockage induces hypoxia, and it may directly cause oxidative imbalance by producing reactive oxygen species and triggering an inflammatory cascade. This condition could develop endothelial dysfunction, resulting in developing cardiovascular and cerebrovascular diseases. Furthermore, this phenomenon occurs more severely in longer respiratory events than in shorter events, most probably due to the higher hypoxic burden associated with longer respiratory events.

3. At the conclusion of the introduction, provide a more detailed statement of the study's precise hypotheses or aims.

Thank you for your comments. We have provided the study’s detailed aims.

(Lines 83-88) Therefore, in this study, we aimed to address the constraints observed in previous studies by focusing on several individuals diagnosed with OSA through overnight PSG and accurate pure-tone audiometry assessing from 0.25 to 8 kHz. Thus, by selecting participants with proper diagnosis and strict criteria, we believe that in this study, we could identify the true relationship between OSA and HL. We also aimed to determine which OSA characteristics affect hearing loss using PSG parameters.

Methods:

- Give more information about the inclusion and exclusion criteria as well as patient recruitment.

Thank you for your comments. We have provided the detailed inclusion/exclusion criteria and patient recruitment. This was a retrospective chart-review study; therefore, participants were not recruited, but they voluntarily visited the hospital due to their symptoms.

(Lines 91-103) This study included patients who underwent simultaneous overnight PSG and pure-tone audiometry within 1 year between February 2014 and November 2023. These are patients who voluntarily visited the hospital for diagnosis because they were suspected of having OSA. However, we excluded patients who were not diagnosed with OSA due to unmet criteria, such as an apnea-hypopnea index (AHI) < 5. Patients who underwent pure-tone audiometry due to pathological conditions like sudden hearing loss, otitis media, and trauma were also excluded. Cases in which pure-tone audiometry did not include high-frequency testing or those in which portable PSG was used were also excluded.

Furthermore, to perform a 1:1 age-sex matching with data from the Korean National Health and Nutrition Examination Survey (KNHANES), patients outside the age range of 40 to 80 years were not considered. Overall, 200 participants were initially reviewed, and based on the inclusion and exclusion criteria, 90 patients were enrolled and included in the OSA group.

- Justify the selection of 90 OSA patients as the sample size.

Thank you for your comments. This was a retrospective chart review study; therefore, we did not select the sample size. However, we tried to include as many participants as possible since larger sample sizes could reduce the sampling errors and provide a more accurate representation of the population. We have added a statement indicating that it was a retrospective chart review study at the beginning of the paragraph.

(Line 91) This is a retrospective chart review study.

- Give a more thorough explanation of the statistical analysis, focusing on the multiple regression method.

Thank you for your comments. We have added detailed statistical methods.

(Lines 162-168) The dependent variables were set as PTA and HF PTA.

Furthermore, to investigate the impact of PSG parameters on hearing and adjust for other factors, the independent variables included age, sex, and PSG parameters such as AHI, lowest O2 saturation, maximum apnea length, and maximum hypopnea length. Additionally, physical measurements such as BMI, NC, AC, and HC were included as independent variables. The stepwise selection method was selected for the analysis.

Results:

- Use subheadings to make the results easier to understand and present them in a more structured manner.

Thank you for your valuable comments. We have added the subheadings as follows:

Heading 1: Characteristics of the study population

Subheading: Polysomnography (PSG) parameters, Hearing thresholds

Heading 2: Hearing level according to the presence of OSA

Heading 3: The factors contributing to hearing loss in patients with OSA

Subheading: Differences in PSG parameters between the HL and non-HL groups, Differences in PSG parameters between the HFHL and non-HFHL groups, Factors Associated with hearing loss in patients with OSA

- When applicable, provide confidence intervals in addition to p-values.

Thank you for your kind advice. We have tried to provide confidence intervals to the regression analysis data in Table 4. However, all current regression data are standardized values, and if confidence intervals are displayed, they are converted to non-standardized values. Therefore, the influence of actual independent variables on dependent variables cannot be accurately expressed. Thus, statisticians have recommended that it is better not to display confidence levels along with p-values to ensure clarity.

As shown below, we have prepared two types of tables (the existing one with standardized values and the revised one with unstandardized values with confidence intervals indicated). We would like to select one according to the reviewer's choice. Thank you again for your advice.

| **Dependent variables**  **Independent variables** | | **PTA** | | **HF PTA** | |
| --- | --- | --- | --- | --- | --- |
|  |  | **ß** | **P-value** | **ß** | **P-value** |
| **Age** | | 0.584 | **<0.001** | 0.602 | **<0.001** |
| **Sex** | |  | .587 |  | .326 |
| **PSG parameter** | **AHI** |  | .859 |  | .337 |
|  | **Lowest O2 saturation** | 0.296 | **0.023** | 0.273 | **0.029** |
|  | **Apnea max. length** | 0.404 | **0.002** | 0.425 | **0.001** |
|  | **Hypopnea max. length** |  | .098 |  | .277 |
| **Physical measurement** | **BMI** |  | .383 |  | .395 |
|  | **NC** |  | .576 |  | .550 |
|  | **AC** |  | .741 |  | .778 |
|  | **HC** |  | .113 |  | .175 |

| **Dependent variables**  **Independent variables** | | **PTA** | | **HF PTA** | |
| --- | --- | --- | --- | --- | --- |
|  |  | **ß**  **(95% CI)** | **P-value** | **ß**  **(95% CI)** | **P-value** |
| **Age** | | 0.729 (0.521 to 0.938) | **<0.001** | 0.989 (0.724 to 1.254) | **<0.001** |
| **Sex** | |  | .587 |  | .326 |
| **PSG parameter** | **AHI** |  | .859 |  | .337 |
|  | **Lowest O2 saturation** | 0.476 (0.067 to 0.886) | **0.023** | 0.580 (0.061 to 1.099) | **0.029** |
|  | **Apnea max. length** | 0.278 (0.101 to 0.454) | **0.002** | 0.385 (0.161 to 0.608) | **0.001** |
|  | **Hypopnea max. length** |  | .098 |  | .277 |
| **Physical measurement** | **BMI** |  | .383 |  | .395 |
|  | **NC** |  | .576 |  | .550 |
|  | **AC** |  | .741 |  | .778 |
|  | **HC** |  | .113 |  | .175 |

- Take into account include a graph illustrating the connection between the length of apnea and hearing loss.

Thank you for your precious comments. We have added a description and a graph showing the relationship between apnea maximum length and hearing levels.

(Lines 261-263) Pearson correlation analysis showed that apnea maximum length had a correlation coefficient (r) of 0.278 and 0.317 with PTA and HF PTA, respectively (Fig 3).

**B**

**A**

**Fig 3.** The correlation between apnea maximum length and hearing level

(A) Apnea maximum length showed a moderate correlation with PTA (r=0.278).

(B) Apnea maximum length showed a stronger correlation with HF-PTA (r=0.317).

PTA, pure-tone average; HF PTA, high-frequency pure-tone average

Discussion:

- Talk about the findings' clinical implications in greater detail.

Thank you for your comments. We have added the following paragraph to the discussion.

(Lines 275-283) In this study, we aimed to investigate the association between OSA and HL and determine factors influencing HL. Our findings suggest that OSA is associated with HL, highlighting the need to give closer attention to hearing levels in patients with OSA. The significant impact of apnea duration on hearing loss, even after controlling for other factors, indicates that prolonged apnea episodes may damage the auditory system. Clinically, this underscores the importance of diagnosis and treatment of OSA, particularly in the early stages, to potentially prevent further hearing decline. These findings suggest that incorporating a greater focus on hearing level in OSA management strategies could be crucial for enhancing the overall quality of care and preventing further complications associated with hearing loss in patients with OSA.

- Make a thorough comparison and contrast between the findings and earlier research on OSA and hearing loss. cite doi:10.12659/msm.897347.

Thank you for your comments. We have modified the paragraphs and cited the recommended article.

(Lines 296-324) Furthermore, this study included all frequency and high-frequency assessments due to the varying results of previous studies. Notably, some studies have claimed that OSA causes HL at all frequencies, whereas others have reported HL only at high frequencies. Kayabasi et al. proposed that this difference was due to the severity of OSA. They compared the hearing levels in 120 patients with OSA and found that although patients with moderate OSA showed hearing loss only at high frequencies, those with severe OSA showed hearing loss at all frequencies. Conversely, other studies claimed that the effect of snoring on hearing loss was observed at high frequencies rather than hypoxic damage due to OSA. Ekin et al. compared the hearing levels between patients with OSA, the simple snoring group, and normal controls. They found no difference in hearing levels between patients with OSA and the simple snoring group; however, the simple snoring group exhibited HL in extended high frequencies > 10 kHz compared with the other two groups. Based on the results, they argued that snoring, which was a continuous noise exposure, was the main cause of hearing loss, especially at high frequencies. However, the participants in this study were mostly those with moderate OSA; therefore, there may not have been much difference in their hearing levels compared with the normal control group. Furthermore, we did not consider OSA severity or snoring in the present study since we have focused more on strictly selecting participants and controlling other factors that may affect hearing levels. Therefore, future studies that consider OSA severity and snoring should further explore the relationship between OSA and hearing level.

In this study, the OSA group exhibited significantly worse hearing across all frequency ranges; however, no statistical significance was observed at 4 kHz. This is likely because the present study was conducted in Korea, where several years of military service are mandatory. Noise-induced hearing loss associated with military service is frequently reported in Korean men, leading to substantial HL, particularly at approximately 4 kHz [27]. Therefore, considering the high proportion of men in this study, this additional noise effect might have contributed to the reduced statistical differences at this frequency. Consequently, our result confirmed that hearing levels were significantly worse across all frequencies in the OSA group compared with the normal control.

.

- Go into further detail about the study's limitations, including any potential confounding variables that were overlooked.

Thank you for your comments. We have added the potential confounding variables to the article.

(Lines 371-373) Notably, given reports of various chronic conditions, including cardiovascular disease, hypertension, and Diabetes mellitus being associated with OSA and HL, the failure to consider these factors could also be viewed as a limitation.

- Make more precise suggestions for future lines of inquiry.

Thank you for your comments. We have added the plans for future studies.

(Lines 313-315) Therefore, future studies that consider OSA severity and snoring should further explore the relationship between OSA and hearing level.

(Lines 388-399) However, future studies should address and overcome these limitations while exploring parameters capable of effectively illustrating the relationship between OSA and HL. Specifically, potential confounding variables, such as snoring sound, lifestyle factors, or additional comorbidities that may influence OSA severity and hearing, should be considered.

Moreover, recent attention has been drawn to the concept of ‘Hypoxic burden’, which refers to the cumulative impact of oxygen deprivation during sleep. Therefore, investigating the relationship between hypoxic burden and hearing loss could provide valuable insights into how intermittent hypoxia may contribute to auditory damage in patients with OSA. Thus, further exploration of this parameter may offer a more comprehensive understanding of the mechanisms driving the association between OSA and HL, as well as potential therapeutic targets.

Conclusion

- Rewrite the conclusion to make it more impactful and succinct.

- Make a clearer emphasis on the study's significance and main innovative findings.

Thank you for your comments. We have modified that paragraph as follows:

(Lines 380-388) However, despite its limitations, this study provides valuable insights into the relationship between OSA and hearing loss, utilizing overnight PSG and precise pure-tone audiometry ranging from 250 to 8 kHz. The results clearly show that individuals with OSA have poorer hearing across all frequencies compared with those without OSA, and a significant correlation was found between apnea duration and hearing loss. Notably, this study highlights the potential of using apnea duration as an indicator of hypoxia-related auditory damage. These findings underscore the importance of early detection and intervention in patients with OSA to prevent further hearing loss, marking a crucial step toward understanding the complex link between sleep apnea and hearing impairment.

**Reviewer #2**

Very good work on controlling for factors with adequate matching. Including a low-risk SBQ result in the control group as a criterion for acceptance shows the robustness of the methodology.

The conclusion of the abstract is misleading, which findings underscore the HL? The research findings underscore? or is the previously suspected HL on the OSA patients what is underscored? I recommend rewording this concept.

**Response:**

Thank you for your valuable comments. We agree that the current expression is confusing. We have modified the conclusion of the abstract as follows.

(Lines 38-41) These findings underscore the significant association between OSA and reduced auditory function, with apnea duration standing out as a crucial factor contributing to hearing loss. Our results suggest that prolonged apnea duration may be a marker of chronic hypoxic damage in patients with OSA, further clarifying its potential role in the development of hearing loss.
